# Estimating RNA dynamics using one time point for one sample in a single-pulse metabolic labeling experiment

**DOI:** 10.1186/s12859-022-04672-4

**Published:** 2022-04-22

**Authors:** Micha Hersch, Adriano Biasini, Ana C. Marques, Sven Bergmann

**Affiliations:** 1 Department of Computational Biology, University of Lausanne, Lausanne, Switzerland; 2 Swiss Institute of Bioinformatics, 1015 Lausanne, CH Switzerland; 3 Present Address: RNA Therapeutics Institute, University of Massachusetts Medical School, Worcester, MA USA

**Keywords:** RNA metabolic labeling, RNA dynamics, RNA responsiveness, Zeisel model

## Abstract

**Background:**

Over the past decade, experimental procedures such as metabolic labeling for determining RNA turnover rates at the transcriptome-wide scale have been widely adopted and are now turning to single cell measurements. Several computational methods to estimate RNA synthesis, processing and degradation rates from such experiments have been suggested, but they all require several RNA sequencing samples. Here we present a method that can estimate those three rates from a single sample.

**Methods:**

Our method relies on the analytical solution to the Zeisel model of RNA dynamics. It was validated on metabolic labeling experiments performed on mouse embryonic stem cells. Resulting degradation rates were compared both to previously published rates on the same system and to a state-of-the-art method applied to the same data.

**Results:**

Our method is computationally efficient and outputs rates that correlate well with previously published data sets. Using it on a single sample, we were able to reproduce the observation that dynamic biological processes tend to involve genes with higher metabolic rates, while stable processes involve genes with lower rates. This supports the hypothesis that cells control not only the mRNA steady-state abundance, but also its responsiveness, i.e., how fast steady state is reached. Moreover, degradation rates obtained with our method compare favourably with the other tested method.

**Conclusions:**

In addition to saving experimental work and computational time, estimating rates for a single sample has several advantages. It does not require an error-prone normalization across samples and enables the use of replicates to estimate uncertainty and assess sample quality. Finally the method and theoretical results described here are general enough to be useful in other contexts such as nucleotide conversion methods and single cell metabolic labeling experiments.

**Supplementary Information:**

The online version contains supplementary material available at 10.1186/s12859-022-04672-4.

## Introduction

Since the advent of molecular biology, a consensus has emerged that the regulation of gene expression underlies most biological processes including development, disease and adaptation [1–3]. While gene expression regulation has mostly been associated with activating the production of RNA (e.g. through transcription factors), it has become apparent that the regulation of RNA splicing and RNA stability also plays an important role in determining the expression level of a gene [4, 5]. Taking advantage of high throughput RNA quantification protocols, methods designed to distinguish the effects of RNA synthesis, processing and degradation at the transcriptome-wide level have been developed. Among them, RNA metabolic labeling techniques relying on chemically modified ribonucleotides such as 6-thianoguanosine (6sG) 4-thiouridine (4sU), 5’-Bromouridine (BrU) or 5-ethynyluridine (EU) have been widely adopted (as reviewed in [6]), due to their minimal impact on cellular function [7, 8]. Briefly, incubating cells with modified ribonucleotides for a limited period of time (referred to as the pulse), and their concomitant incorporation in newly synthesized transcripts, allows distinguishing newly transcribed from preexisting RNA, which can be biochemically separated and quantified. The separation can be performed using thiol-specific biotinylation and streptavidin-dependent enrichment of biotinylated RNA [9] or, through a more recent improvement, by direct capture of 4sU onto a solid phase using a methane thiosulfonate resin [10]. Following the quantification, which was based initially on microarray technologies [11] and now on RNA-seq [12, 13], the resulting data can then be used to estimate RNA decay rates. More recently, methods that rely on nucleotide conversion have been used to the same effect, with the advantage of circumventing the cumbersome biochemical enrichment and separation step: SLAM-Seq chemo-selectively labels 4sU with iodoacetamide to enable the *in silico* identification of 4sU containing RNA. While this method avoids the biases arising from the enrichment-based methods described above, it also has disadvantages. First it requires higher RNA sequencing depth for quality control [14] and more resources to implement. Second, SLAM-DUNK, the currently available software for analyzing SLAM-Seq data is only compatible with a modified 3’end mRNA sequencing method, a specialized approach which is only applicable to a fraction of all RNAs present in a cell [15].

In contrast, several methods to estimates RNA dynamics from metabolic labeling experiment data have been developed [16–18] (see [19] for a review). Typically, labeled transcript abundances are fitted to an exponential function approaching to steady-state equilibrium during the labeling pulse (or after the pulse, during the so-called chase phase when labeled transcripts are being depleted). The RNA half-life can then be estimated from those exponential fits [20–22]. This requires time-course experiments in order to have enough points for fitting, as well as a way to normalize RNA concentrations across samples, either using spike-ins [23], or using internal controls such as intron concentrations [24]. The INSPEcT method [25] goes beyond first order dynamics and takes into account the RNA processing rates, which are estimated along with the degradation and synthesis rates. This method works by first estimating rates for individual samples by assuming, by default, no degradation during the pulse and then uses those estimates as a starting point for fitting models of rate evolution for all the rates of all samples. Those methods rely, for each sample, on a the separate quantification of labeled RNA on one hand and of total (mixed labeled and unlabeled) and/or unlabeled (or pre-existing) RNA on the other hand. In its later version, INSPEcT was extended to estimate rates without labeling the sample [26].

In this work, we build on the INSPEcT approach and derive an exact solution (when it exists) for the initial rate estimates without making the assumption of no labeled transcript degradation. This is achieved by considering the intron to exon ratio for each transcript in both the labeled and unlabeled RNA pools, thus allowing to bypass the need for normalization across those two samples. We can thus infer synthesis, processing and degradation rates from a single sample and time point. Those rates can be used as such, allowing to reduce the experimental load and costs and compare rates across samples and time points. But they can also be used, as in INSPEcT, as initial estimates for mutliple sample-based rate estimation. Applying our method to our own experimental data and using a single sample and time point, we obtain synthesis and processing rates that are well correlated with the ones obtained using INSPEcT first guess. The degradation rates, on the other hand, correlate poorly across the two methods, but those computed with our method correlate better than INSPEcT with previously published mRNA degradation rates obtained with three replicates and seven time points in a nuclear conversion protocol [27]. Because it can be reduced to numerically solving an equation with a single unknown on a bounded domain, it is also much faster than INSPEcT. Moreover, our results are consistent with an adapted gene-specific mRNA responsiveness and co-transcriptional mRNA processing [28].

## Method

### Overview

**Fig. 1:**
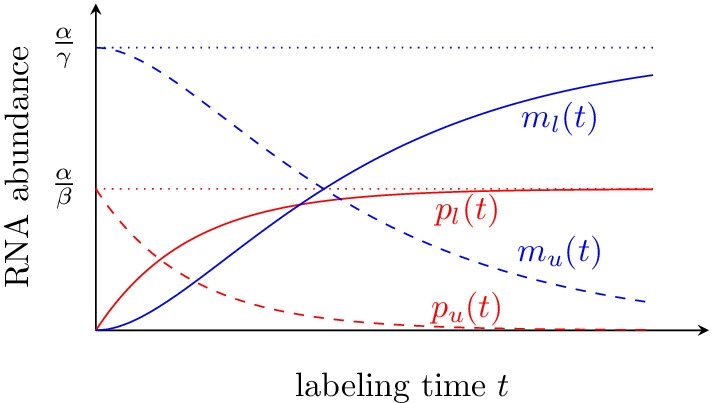
Evolution of unlabeled and labeled, premature and mature RNA during labeling according to the Zeisel model. Dotted horizontal lines correspond to steady-state levels, dashed lines correspond the unlabeled RNA and solid lines to labeled RNA. Processing and degradation rates can be estimated from the ratios of the two dashed lines and of the two solid lines at a single time point

This paragraph summarizes the general strategy of the method, with references to relevant equations indicated in parentheses. We use the Zeisel model of RNA dynamics [29] to model both the unlabeled and the labeled RNA (1, 2). Using the standard procedure for solving systems of linear differential equations, we find its general solution and its free parameters by setting the initial conditions for both the unlabeled (or pre-existing) and the labeled RNA (3–6), as illustrated in Fig. 1. We can then express, for a given gene and for both unlabeled and labeled RNA, the ratios of intron to exon expression level as functions of the processing and degradation rate of that gene (8,9). These two ratios are independent from the RNA synthesis rate. Using the intron to exon ratios as observables, we are left with two non-linear equations and two unknowns, namely the processing and degradation rates. These equations are then reparametrised with dimensionless parameters and reduced to a single non-linear equation with one unknown (13). This resulting equation is only defined on a bounded domain (14). Our rates can thus be inferred by numerically solving that equation on a bounded domain, which is very fast. In addition, we prove in Additional file 3: Appendix C that this equation, under certain conditions, has a single solution (but in general it can also have two or no solution).

### Model

Like previous work [25], we use the Zeisel model of RNA synthesis, processing and degradation [29].

1 $$\begin{aligned} \dot{p}&= \alpha -\beta p\end{aligned}$$

2 $$\begin{aligned} \dot{m}&= \beta p- \gamma m\,, \end{aligned}$$

where $p$ is the premature RNA, $m$ the mature RNA, and $\alpha$, $\beta$, $\gamma$ are RNA the synthesis, processing and degradation rates. This model can be solved analytically (see Additional file 1: Appendix A). In particular, enforcing the boundary conditions corresponding to the unlabeled RNA, namely that it is at steady state when the pulse starts (*t* = 0) and that subsequently no more pre-mature RNA is produced, results in

3 $$\begin{aligned} p_{u}(t)&= \frac{\alpha }{\beta }\exp (-\beta t) \end{aligned}$$

4 $$\begin{aligned} {m_{u}}(t)&= \frac{\alpha }{\gamma -\beta }\exp (-\beta t) - \frac{\beta \alpha }{\gamma (\gamma -\beta )}\exp (-\gamma t)\,, \end{aligned}$$

where the *u* subscript indicates that this corresponds to the unlabeled RNA pool.

Enforcing boundary conditions corresponding to the labeled RNA, namely that it is not (yet) expressed at $t=0$ leads to

5 $$\begin{aligned} p_{l}(t)&= \frac{\alpha }{\beta }\big (1 - \exp (-\beta t)\big ) \end{aligned}$$

6 $$\begin{aligned} m_{l}(t)&= \frac{\alpha }{\gamma }\left( 1+\frac{\beta }{(\gamma -\beta )}\exp (-\gamma t)\right) - \frac{\alpha }{\gamma -\beta }\exp (-\beta t) \end{aligned}$$

where the *l* subscript indicates that this corresponds to the labeled RNA pool.

### Inferring synthesis, processing and degradation rates

We consider that the exonic RNA abundance $\chi$ corresponds to the premature and mature RNA, while the intronic RNA abundance $\iota$ correspond to the premature RNA only. Furthermore, we assume that $\chi$ and $\iota$ are suitably normalised for exonic and intronic length so that they are proportional to the number of transcripts. We can then compute:

7 $$\begin{aligned} \frac{\iota }{\chi } = \frac{p(T)}{p(T)+m(T)}\,, \end{aligned}$$

where *T* is the labeling time.

In the case of unlabeled fraction, we have

8 $$\begin{aligned} \frac{\iota _{u}}{\chi _{u}}&= \frac{p_{u}(T)}{p_{u}(T)+{m_{u}}(T)}\nonumber \\&= \frac{E_\beta }{\beta \big ((\frac{1}{\beta }+\frac{1}{\gamma -\beta })E_\beta -\frac{\beta }{\gamma (\gamma -\beta )}E_\gamma \big ) }\nonumber \\&= \frac{E_\beta }{\frac{\gamma }{\gamma -\beta }E_\beta -\frac{\beta ^{2}}{\gamma (\gamma -\beta )}E_\gamma }\nonumber \\&= \frac{(\gamma -\beta )E_\beta }{\gamma E_\beta -\frac{\beta ^{2}}{\gamma }E_\gamma }\nonumber \\&= \frac{\gamma (\gamma -\beta )E_\beta }{\gamma ^{2}E_\beta -\beta ^{2}E_\gamma }\, \end{aligned}$$

where we define $E_\beta =\exp (-\beta T)$ and $E_\gamma =\exp (-\gamma T)$ as abbreviations.

For the labeled fraction, we have

9 $$\begin{aligned} \frac{\iota _{l}}{\chi _{l}}&= \frac{p_{l}(T)}{p_{l}(T)+m_{l}(T)}\nonumber \\&= \frac{\big (1 - E_\beta \big )}{\big (1 - E_\beta \big )-\frac{\beta }{\gamma -\beta }E_\beta + \frac{\beta }{\gamma }\big (1+ \frac{\beta }{\gamma -\beta }E_\gamma \big )}\nonumber \\&= \frac{\big (1 - E_\beta \big )}{\frac{\gamma +\beta }{\gamma }-\frac{\gamma }{\gamma -\beta }E_\beta +\frac{\beta ^2}{\gamma (\gamma -\beta )}E_\gamma }\nonumber \\&= \frac{\gamma (\gamma -\beta )\big (1 - E_\beta \big )}{\gamma ^{2}-\beta ^{2}+\beta ^{2}E_\gamma -\gamma ^{2}E_\beta }\nonumber \\&= \frac{\gamma (\gamma -\beta )\big (1 - E_\beta \big )}{\gamma ^{2}\big (1-E_\beta \big )-\beta ^{2}(1-E_\gamma \big )}. \end{aligned}$$

We notice that this last expression is of the same form as the one for the unlabeled fraction (8), but replacing exponentials by their complement to one. Importantly these two fractions do not depend on $\alpha$, which (unlike [26]) allows our method to estimate processing and degradation rates independently from the synthesis rate.

Denoting $a= \frac{\iota _{u}}{\chi _{u}}$ and $b= \frac{\iota _{l}}{\chi _{l}}$ as the observable unlabeled and labeled ratios of intron to exon abundances, we are left with a system of two equations and two unkowns $\beta$ and $\gamma$, which we now set out to solve. First, we reparameterize our system with $\beta = k\gamma$ and define $E_{k\gamma }=E_\beta =\exp (-k\gamma T)$ leading to

10 $$\begin{aligned} a&= \frac{(1-k)E_{k\gamma }}{E_{k\gamma }-k^{2}E_\gamma } \end{aligned}$$

11 $$\begin{aligned} b&= \frac{(1-k)\big (1-E_{k\gamma }\big )}{\big (1-E_{k\gamma }\big )-k^{2}\big (1-E_\gamma \big )}\,. \end{aligned}$$

It is shown in Additional file 2: Appendix B that this system of equations can be simplified to the following system of equations where $k$ is isolated.

12 $$\begin{aligned} \log \left( \frac{b-a}{a(bk+b-1)}\right)&= k\gamma T \end{aligned}$$

13 $$\begin{aligned} \frac{k}{k-1}\log \left( \frac{k+a-1}{k^{2}a}\right) -\log \left( \frac{b-a}{a(bk+b-1)}\right)&= 0 \,, \end{aligned}$$

with the following domain of definition ${\mathcal {D}}$ for $k$:

14 $$\begin{aligned} \max \left( \frac{1}{b}-1,1-a\right)< k < \frac{1}{a}-1\,. \end{aligned}$$

The above equation (13) does not explicitely depend on *T* and can be solved numerically on ${\mathcal {D}}$. In practice $a$ and $b$ are approximated by $r_{u}$ and $r_{l}$, defined as the length-normalized intronic to exonic read count ratio (or TPM ratio) for the unlabeled and for the labeled sampled respectively.

We further prove in Additional file 3: Appendix C that for $b>\frac{1}{2-a}$, Eq. (13) has a single solution in the domain given by (14), which can be found very efficiently. This enables the estimation of the processing and degradation rates for a single sample. Moreover, since the reduced equation is independent from *T*, uncertainty on its true value does not affect the relative values of the rate estimates. Hence replicates can be used to assess the reliability of the estimates and time courses allow to test whether the rates are constant as assumed by the model.

If (13) does not have a solution, estimates can be obtained by minimizing (in log space) the squared Euclidean distance between the observed (i.e., $r_{u}$, $r_{l}$) and derived values of $a$ and $b$:

15 $$\begin{aligned} f(k,\gamma T) =&\left( \log (r_{u}) - \log \left( \frac{(1-k)}{\exp (-k\gamma T)-k^{2}\exp ((k-1)-\gamma T)}\right) \right) ^2 \nonumber \\&+\left( \log (r_{l}) -\log \left( \frac{(1-k)\left( 1 - \exp (-k\gamma T)\right) }{\left( 1-\exp (-k\gamma T)\right) -k^{2}(1-E_\gamma \big )} \right) \right) ^{2}\,. \end{aligned}$$

The ratios $r_{u}$, $r_{l}$ must be smaller than one to make sense within our model and genes where this is not the case should be discarded. The log function is used to give exon and intron counts equal standing.

The above bivariate function can be reduced to a univariate function $f^{*}$ using (12):

16 $$\begin{aligned} f^{*}(k) = f\left( k,\frac{1}{k}\log \left( \frac{r_{l}-r_{u}}{r_{u}(r_{l}k+r_{l}-1)}\right) \right) \end{aligned}$$

The processing and degradation rates are derived from $k$ using (12) where $a$ and $b$ are again approximated by $r_{u}$ and $r_{l}$ respectively. Then the synthesis rate $\alpha$ can be easily obtained from (4), where ${m_{u}}$ is approximated by $\chi _{u} - \iota _{u}$ as unlabeled RNA is likely more precisely quantified, due to the usual presence of some unlabeled RNA in the labeled RNA pool (captured through unspecific binding):

17 $$\begin{aligned} \gamma = \frac{1}{kT}\log \Big (\frac{r_{l}-r_{u}}{r_{u}(r_{l}k+r_{l}-1)}\Big ) \quad \quad \beta =k\gamma \quad \quad \alpha = \frac{\gamma (\gamma -\beta )(\chi _{u}-\iota _{u})}{\gamma E_\beta - \beta E_\gamma } \end{aligned}$$

Note, that the estimation of $\alpha$ using the labeled RNA and (6) is also possible, see Additional file 4: Fig. D8 in Appendix D for a comparison.

## Results

### Simulated data

In order to confirm that our method can be applied in principle, we evaluated our method on simulated data, where the data was generated using the exact model used to develop the method (see equations 3 and following). As a first step, we did not simulate noise in the model so as to validate the mathematical developments above and our implementation of the method. We generated 50000 random value for $\alpha$, $\beta$, and $\gamma$ ranging between $\exp (-5)$ and $\exp (5)$ and computed the corresponding values for $\iota$ and $\chi$. We then computed $r_{u}$ and $r_{l}$ by taking the ratio. Estimates ${\hat{\beta }}$ and ${\hat{\gamma }}$ where then inferred by using $r_{u}$ and $r_{l}$ as an input to the method and compare the original $\beta$ and $\gamma$.

**Fig. 2:**
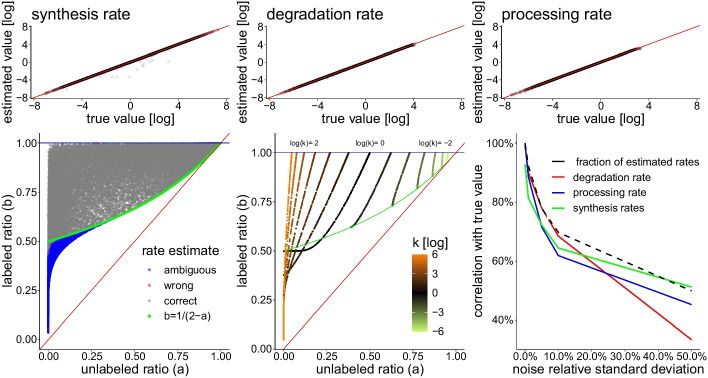
Simulated data. Top row: the method correctly estimates synthesis, processing and degradation rates. Points with ambiguous solutions are not shown. Some points corresponding to high rates cannot be estimated correctly as the system as already reached steady state during the simulated “pulse’. Bottom left: the measurement space can be partitioned into ambiguous and unambiguous regions. The green line corresponds to $b=\frac{1}{2-a}$. Above that line, rates are correctly and unambiguously estimated. Boundary cases are sometimes wrongly estimated, probably due to numerical errors (red dots). Bottom center: Trajectories in the phase space are solely determined by the *k* parameter. They start at time $T=0$ at the top ($b=1$) and go down. For $k<1$ the trajectories (in olive) remain above the green line defined by $b=(2-a)^{-1}$ and do not cross. For $k>1$ (in orange), they cross each other below the green line. The velocity at which the system follows the trajectory depends on the actual values of $\beta$ and $\gamma$. Bottom right: effect of adding simulated Gaussian noise to the exon and intron quantifications on the correlation between estimated and true values of the rates. The abscissa indicates the standard deviation of the noise relative to the expression value. The black dashed line indicates the fraction of transcripts for which rates could be estimated. For example, a 10% standard deviation for noise, provides rates for about 70% of transcripts with around 62%-68% correlation with the true values

Numerically solving equation (13), yielded either one or two solutions. The results for the unambiguous cases are shown in Fig 2, left. We see that in virtually all cases, the method yields accurate estimates of the processing and degradation rates. For a few points, the method is less accurate at the upper boundary of the parameter space, probably due limited floating point precision. Indeed, if the labeling time is too long with respect to the metabolic rates, virtually all unlabeled RNA are degraded and the rates cannot be reliably estimated.

As we are considering single-sample estimates, it is possible to chart the observable space given by $a$ and $b$ and see when the method provides unambiguous results. Figure 2, bottom left, confirms that for $b>\frac{1}{2-a}$ the method provides a unique (and correct) solution as proven in Additional file 3: Appendix C. Below this line (displayed in green), the methods provides ambiguous results as two distinct set of values $\beta$ and $\gamma$ can account for the same value of $a$ and $b$ (in blue). It is also possible to visualize the trajectories of the observables $a$ and $b$ for various values of *k*, as depicted in Fig. 2, bottom center. When $T=0$, trajectories start from the top of the space at $(\frac{1}{1+k},1)$. When $k<1$, as time passes the system moves down to $(a,b) \rightarrow (1-k,\frac{1}{1+k})$. For $k\ge 1$, trajectories move to $(0,\frac{1}{1+k})$. Note that this is the expected case, as the splicing of mRNA occurs in general faster than its degradation. Note that, in this case, trajectories cross below the green line, explaining why two solutions can be found for a single value of $(a,b)$. The speed at which the system follows these trajectories depends on $\gamma$.

In order test the robustness of the method to noise, we added various levels of Gaussian noise (in log space) to the simulated intron and exon quantifications and compared the resulting rates with their “true” value, using Pearson correlation. The results are shown on the last panel of Fig. 2. We first note that, when adding noise, transcripts that fall above the blue horizontal line or below the red diagonal line of the bottom left panel cannot be estimated as ${\mathcal {D}}$ in (14) is not defined. The fraction of rates that can be estimated drops significantly as the noise increase (see back dashed line). We also see that the degradation rate is less affected by low level of noise, but more affected by a high level of noise compared to the processing rate, while the converse is true for the synthesis rates. A correlation with the true rates above 60% is obtained with a standard deviation of the noise below 10%, which is a reasonable scenario for decently expressed transcripts.

### Real data

In order to assess the performance of the method on real data, we applied our method on the 4sU labeling experiment described in [30]. Briefly, mouse embryonic stem cells were plated at a density of 40,000 cells/$\hbox {cm}^{2}$ on gelatin-coated 10cm tissue culture plates and grown for approximately 14 hours. After addition of 4sU to the growth medium, cells were incubated at 37C for 10 minutes (10 minutes labeling time). RNA was then extracted and processed according to the protocol described in [31]. Reads that did not map to mouse ribosomal RNA sequences were aligned to intronic and exonic sequences (ENSEMBL v91 mus musculus reference) using STAR V2.5 [32] and quantified using RSEM V1.1.17 [33], yielding intron and exon expression levels for unlabeled and labeled RNA for each detected transcript.

**Fig. 3:**
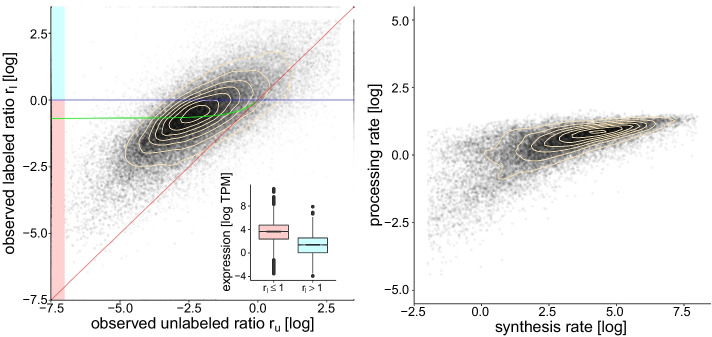
Real data. Left: Each point corresponds to a transcript with its transparency reflecting log expression value. Like in the previous figure, the green line is defined $y=(2-x)^{-1}$. For transcripts lying between the abscissa (in blue) and the green line, estimates of processing and degradation rates can be obtained by solving (13). For transcripts lying between the diagonal (in red) and the green line, estimates can be obtained by minimizing (16). The observed ratios for the remaining transcripts are not coherent with the model and are discarded. These trancripts (above the blue line) are lowly expressed compared to the ones below the blue line (see inset). Right: RNA processing rates are highly correlated to the synthesis rates (73%), which is consistent with co-transcriptional RNA processing. In both plots, density contour lines are shown in beige and axes are cropped for a better view of the data

For a single sample, the observable space represented in Fig. 2 (bottom left and center) is represented (in log coordinates) in Fig. 3, left. We see that, while the points are centered on the expected region of the observable space, many transcripts lie below the diagonal or above the $r_{l}=1$ (or $\log (r_{l})=0$) line (in blue), which is not compatible with our model. We observe that those incompatible transcripts lying above the $r_{l}>1$ line are expressed at a much lower level than the transcripts lying below this line (see inset). A lower signal to noise ratio in low expressed genes could explain this difference, in line with the simulations above. However, another likely explanation pertains to the fact that co-transcriptional processing is not accounted for by the Zeisel model. While it has been documented that an RNA molecule is often processed while being synthesized (the “assembly-line model”) [28], the Zeisel model considers synthesis and processing as two independent point events. This discrepancy is likely to be more relevant for short-lived (and thus low-expressed) transcripts, a sizeable fraction of which is expected to be nascent at sequencing time. Those nascent transcripts may contribute to an intron to exon ratios higher than one when they are incompletely synthesized (for example if the last exon has not yet been produced). This hypothesis is corroborated by considering unspliced transcripts length, which putatively affects synthesis time and thus the probability of being nascent at sequencing time. Transcripts lying above the $r_{l}>1$ line are indeed longer than those lying below this line ($\mathrm {p-value} < 10^{-100}$, Wilcoxon test).

**Fig. 4:**
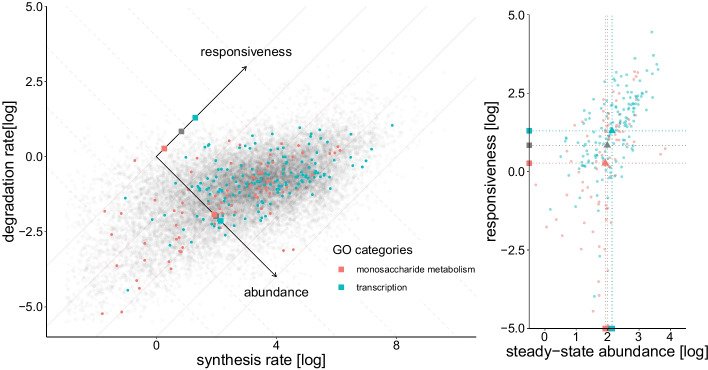
Left: Estimated RNA synthesis and degradation rates obtained from a single sample. These rates can also be considered in a different and maybe functionally more relevant frame of reference defined by the steady-state abundance (first axis) and gene responsiveness (second and perpendicular axis), as illustrated by the background grid. Genes involved in fast adapting biological processes (such as transcription) tend to be more responsive than genes involved in stable functions (such as monosaccharide metabolism). The squares on the axes represent the projections of the mean rates for the respective categories (gray representing genes that belong to neither of the two categories) and indicate that mean transcript responsiveness (but not abundance) is strongly affected by the category. These two GO categories were selected for illustration because they were previously reported to be mostly enriched in high and low turn-over genes respectively [13]. Right: Same data as in left, but rotated and showing only colored dots, for visibility

The transcripts incompatible with our model, amount to 25% of protein-coding genes with an exon TPM higher than 1, and are discarded from further analyses. The processing and degradation rates were computed either by solving (13) when $r_{l}>(2-r_{u})^{-1}$ or by optimizing (16) otherwise. For these cases that had two solutions (6% of the transcripts), we selected the one corresponding to rates most consistent with the other transcripts.

The resulting synthesis and processing rates for protein-coding genes are depicted in Fig. 3, right. Although processing rates span a smaller range of values, they are highly correlated (74%), which is not surprising as RNA processing occurs co-transcriptionally [28]. More remarkable is the correlation of synthesis and degradation rates, displayed in Fig. 4, left. At 62%, it is very similar to the 66% reported by [27] for the same cell type. This is also consistent with the emerging concept of a coupling between RNA transcription and decay [34]. Our data indicate that genes span a large range of dynamics, irrespective of their expression level. Indeed, genes with high synthesis and degradation rates can have the same steady-state expression level as genes with low synthesis and degradation rates. However, the former will reach this steady state faster than the latter. It thus makes sense to consider our RNA metabolic rates in the functional frame of reference indicated in Fig. 4, left. One axis corresponds to the steady-state RNA abundance, given by the log-ratio of synthesis over degradation rates (or equivalently by the difference of log of the rates). The second axis corresponds to the responsiveness of the gene, i.e. how fast it reaches steady state (computed by the sum of the log of the synthesis and degradation rates). It has been observed before that genes involved in more reactive and dynamic biological processes such as chromatin remodeling or transcription regulation tend to have a higher turnover than genes involved in more stable processes such as basic metabolism [13]. We checked that our data confirm this observation by looking at the Gene Ontology (GO, [35]) annotations of biological processes most associated by [13] with high and low turnover, namely “transcription” and “monosaccharide metabolism”. Despite having similar steady-state abundances, transcripts of genes involved in transcription indeed have significantly faster dynamics and the ones involved in monosaccharide metabolism have significantly slower dynamics than the rest of the genes, as illustrated by the squares in Fig. 4, left and right. Other categories where our data confirms faster genes include chromatin modifications, cell cycle and transcription regulation.

**Fig. 5:**
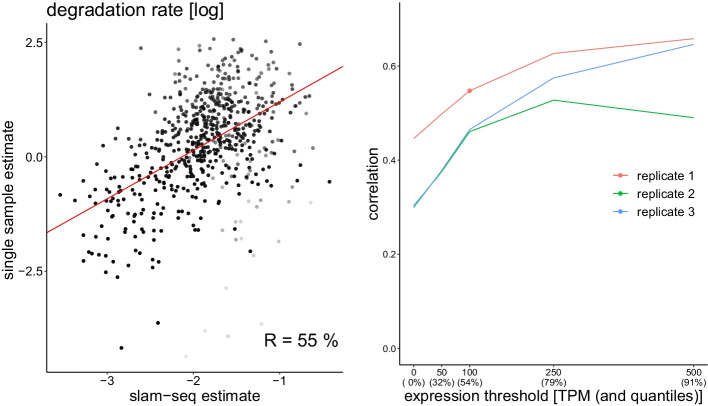
Left: degradation rates estimated from a single sample plotted against degradation rates published in [27] (obtained using slam-seq). The red line is obtained through weighted linear regression. The weights are set as $1-r_{l}$ as indicated by the transparency of the dots. The (weighted) correlation of 55% indicates that the estimated rates are meaningful. Only genes with a mean exon TPM above 100 are taken into account. Right: Correlation between degradation rates obtained by [27] and the ones obtained our single-sample method as a function of expression level. Each line represents a biological replicate. The red dot corresponds to the data shown on the left. As expected, the correlation is higher for highly expressed genes, as the intro to exon ratios can be more reliably estimated. In this experiment, replicate 1 correlates better than the two others, indicating that it is probably of better quality

We assessed the precision of our method by comparing the resulting degradation rates to those published for the same cell type by [27]. Those were obtained by using three replicates and seven time points and applying the SLAM-seq nucleotide-conversion method that, unlike metabolic labeling, does not require biochemical separation between the labeled and unlabeled RNA and is thus not affected by noise generated by the imperfect separation process (although that method has its own source of noise). From our data, we obtained gene degradation rates by taking, for each gene, the weighted average degradation rates of the corresponding transcripts The weights were given by the mean exonic expression levels (unlabeled and labeled). We expect a lower precision for transcripts close to the $r_{l}=1$ line, for which the labeling time was likely somewhat too short, so to assess the correlation, we weighted the transcripts by $1-r_{l}$. Figure 5, left, compares degradation rates obtained in our experiments with those reported by [27], keeping only genes with an average expression value higher than 100 TPM. We expect a higher precision for highly expressed genes, as this allows for a more precises estimates of the intron to exon ratios. This is indeed the case, and depending on the expression threshold and the sample, the correlation between our data and the previously published rates, we obtain a correlation ranging between $30\%$ and $67\%$ for a single sample estimate (see Fig. 5, left). As these experiments were performed in different labs using different methods, these numbers show that our rates obtained on a single sample and time point are meaningful. For comparison, [36] report correlations around 70% by using the *same* data, but changing only the method of analysis. Using three replicates, [31] report a 26% correlation using the INSPEcT package.

### Comparison with INSPEcT

**Fig. 6:**
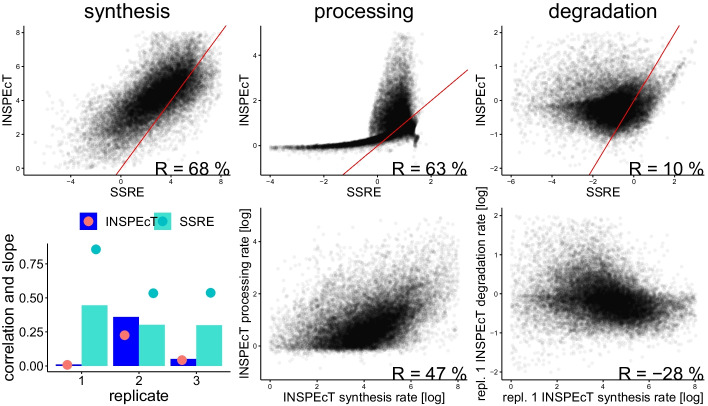
Comparison of our method (SSRE) with the INSPEcT “first guess” on th same data. Top row: direct comparison (in log space) of rates obtained with our method and with the INSPEcT package on a single sample. Synthesis and processing rates are well correlated but not the degradation rate (Spearman correlation shown). The red bar indicates the diagonal. Bottom left: bars indicate the correlation of degradation rates with previously published data [27], as in Fig. 5. The INSPEcT method provides degradation rates with good correlation only for one of the three replicates (repl. 2), whereas it is the case for all three replicates using our method. The big dots indicate the slope of the regression line in log-log space (as in Fig. 5, left). Slopes obtained from SSRE estimates are closer to one, which correspond to the ideal case of a linear relationship between the (non-log) rates. Bottom center and right: Rates obtained with INSPEcT also reproduce the positive correlation between synthesis and processing rates, but they produce a negative correlation between synthesis and degradation rates, unlike our method (see Fig. 4, left) and previously published results. [27]

Since our method estimates metabolic rates from a single sample, we decided to compare its results to the “initial guess” provided by the INSPEcT method, to our knowledge the only other method that does not need multiple samples. Note, however, that those rates are only the initial step of the INSPEcT method, and should not be confused with the global outcome of INSPEcT, which then aggregates multiple samples for the estimation. For concision, we will in the section refer to our method as SSRE (Single Sample Rate Estimation). The main differences between the two approaches is that INSPEcT assumes no degradation on labeled RNA and requires the estimation of a scaling factor accounting for the difference in RNA concentration between labeled and unlabeld samples, which SSRE avoids by considering the intron to exon ratio in each sample separately for each sample. Furthermore, INSPEcT requires the estimation the time derivative of the RNA abundances, which is avoided in SSRE by taking advantage of the analytical solution to the Zeisel model.

We used the INSPEcT package for R on the same data from [30] analyzed in the previous sections (see Figs. 3, 4, 5). The same transcript quantification (TPM) used for our estimates was fed into the newINSPEcT function with parameter preexisting=TRUE and then to the ratesFirstGuess function of the INSPEcT package. The expression variance required by this package was estimated from the expression level from all three replicates using Loess regression on the expression level. It took about 20 minutes to estimates rates for each replicate (about 40,000 transcripts) using a single 2GHz core from a laptop computer, whereas our implementation of SSRE (also in R) took about 30 seconds to complete on the same machine. As described in Table 1, SSRE is about 40 times faster than INSPEcT (0.8 ms vs 34 ms per transcript) but there are also more transcripts that it cannot process.

In addition to direct rate comparison, we decided to compare the methods using three criteria: (1) correlation with published rate, (2) rate distributions and (3) reproducibility across replicates. Figure 6 shows that the two methods provides synthesis and processing rates that are well correlated, while degradation rates are not. Moreover, the degradation rates obtained by INSPEcT correlate well to previously published rates only for one of the three samples. In contrast to degradation rates obtained with SSRE, the correlation with previously published rates does not improve when focusing on highly expressed genes, it even become negative for replicates 1 and 3 (data not shown). This suggests that for SSRE, degradation rate estimation is likely to improve with higher sequencing depth (and thus a more precise estimate of the intron to exon ratio). Finally, the rates computed using the INSPEcT method do not exhibit the previously documented correlation between synthesis and degradation rates [27]. This leads us to think that our degradation rates are closer to the real rates than the ones provided by the INSPEcT “first guess”. This should not come as a surprised, as our method does not assume that labeled RNA does not degrade and estimates degradation and processing rates independently from the synthesis rate.

The synthesis and processing rates provided by the two methods are relatively well correlated, and INSPEcT provides rates that are more consistants across replicates (see Supplementary Fig. D.9.) It is also interesting to note that SSRE tends to show an upper bound for the processing rate, while INSPEcT first shows a lower bound for that rate. It is difficult to speculate which (if any) is more likely true, but an upper bound would be consistent with bio-physical constraints in a leaky co-transcriptional RNA splicing setting. Figure D.9 also shows that unlike INSPEcT, SSRE computes degradation rates that span a larger range of values than processing rates, a property also reported in [17] for a different system.

**Table 1:** Comparison of CPU usage and “rejection” rates of INSPEcT and SSRE. The same batch (subset of the data described above) were provided to both methods (implemented in R). The output size refers to the number of transcripts that can be processed by the method and the speed-up is the ratio of INSPEcT over SSRE transcript processing time. SSRE is about 42 times faster than INSPEcT with (0.8 ms vs 34 ms per processed transcript). However, SSRE also discards more transcripts than INSPEcT, providing rates for 38% of them vs 47% for INSPEcT. Those low numbers can be explained by the fact that many transcripts (especially non-coding ones) have a very low expression that is poorly estimated in the labeled RNA pool

Batch size	INSPEcT		SSRE		Speed-up
	Output size	CPU processing time [s] ([ms/transcript])	Output size	CPU processing time [s] ([ms/transcript])	
10	5 (50%)	2.09 (402)	5 (50%)	0.052 (10)	40
1000	469 (47%)	15.889 (33)	387 (39%)	0.310 (0.8)	42
91702	42650 (47%)	1453.336 (34)	34512 (38%)	27.792 (0.8)	42

## Discussion

In this paper, we presented a method to estimate synthesis, processing and degradation rates of RNA transcripts from a single 4sU labeled sample. We validated our method first *in silico* and then on real data obtained from mouse embryonic stem cells. Using our method we first replicated, on a different cell type, previous findings about the enrichment in high or low turn-over genes of specific cellular processes. Second, we showed that the rates obtained with our method correlate well (between 30% and 67%) with published rates obtained by applying SLAM-seq to the same cell types. Methods for such estimation have been published before, but they usually require a sufficient number of samples (around a dozen). We compared our method to the initial step of the INSPEcT method, which handles each sample separately, and obtain similar synthesis and processing rates, but different degradation rates. Our rates correlate more consistently with previously published degradation rates obtained with nuclear conversion methods on the same system, and even more so for highly expressed transcripts. Rates obtained with our method also better reproduce previously observed statistical relationships between rates, although synthesis and processing rates are less consistant across replicates. Taken together these results suggest that our method provides more reliable degradation rates.

In contrast to other methods, our method explicitly uses the analytical solution to the Zeisel model of RNA dynamics. Moreover, our method is self-normalizing as it only uses the ratio of intron to exon expression levels, making it is less affected by differences in sequencing depth of the various samples (although deeper sequencing will provide better estimates). This approach makes our method also faster than other methods as it boils down to numerically solving on a bounded domain either a univariate equation or a one-dimensional optimization for each transcript. Our method could thus be a suitable alternative to the initial step of the INSPEcT method especially when using a large number of samples as it is also about 20 times faster.

Similarly to the initial step of the INSPEcT method, a caveat of our method is that a sizable fraction of mostly lowly expressed transcripts (about 25 % in our case) are inconsistent with the model and their dynamics cannot be estimated. Together with the high correlation between synthesis and processing rate, it suggests that modeling transcription and processing as independent events is a simplification that could be reconsidered, as the coupling between the two has been documented [28]. However, this limitation of the Zeisel model is likely to also affect other methods using it [26, 37].

Another limitation of the method is that, unlike in [26], it does not consider the effect of leakage of unlabeled RNA in the labeled RNA pool because of unspecific capture. This leakage has the effect of reducing $r_{l}$ towards the diagonal, and could potentially be estimated from the data as it is shared across all transcripts. Another improvement would be to embed this method in a probabilistic framework in order to quantify the estimate uncertainty (as in [36] for a simpler model) or to determine the optimal labeling time (as in [38]).

While using a single sample allows to reduce costs, this is not the only merit of this approach. In practice most experiments will have biological replicates, in which case our methods enables obtaining point estimates of $\alpha$, $\beta$ and $\gamma$ for each of them. This in turn allows for estimating their variance, as well as assessing sample quality (e.g. if one of them systematically gives very different estimates for all genes). Moreover, because cell growth is likely to be limited during (short) labeling time, it is less likely to interfere in the estimation process than when using time course data, where it can have an effet [24]. In addition, when used in a time-course experiment with multiple short pulses, our method allows to investigate the evolution of these rates over time and assess whether these rates are stationnary, using tools from time series analysis such as (extended) Kalman filtering. Finally, the theoretical results obtained in this paper, could be used to improve other methods. For example, the method could be used to analyze SLAM-seq data which would reduce the number of samples but also provide estimate for the processing rate. Another possible application is single cell RNA velocity, where the Zeisel model of RNA dynamics is also used, but splicing rates $\beta$ are assumed to be equal for all transcripts [37] or estimated jointly with $\alpha$ and $\gamma$ using an EM algorithm [39]. Our approach involving a reparametrization of the rates using *k* could provide a interesting and computationally cheaper alternative, for example by considering the strong correlation between the synthesis and processing rates. Finally, our method could also be used in conjunction with the recent developements in single cell metabolic labeling experiments [40, 41].

## Supplementary information

**Additional file 1: Appendix A**. Derivation of the model solution.

**Additional file 2: Appendix B**. Equation simplification.

**Additional file 3: Appendix C**. Proof of uniqueness of solution.

**Additional file 4: Appendix D**. Supplementary figures.

## Data Availability

An R package implementing our method is available on github, together with the code used to generate the figures as well as the gene expression data used: https://github.com/BergmannLab/SingleSampleRNAdynamics The raw data files data are available on the Gene Expression Omnibus accession number GEO:GSE150286 (main replicate) and GEO: GSE143277, samples GSM4255969 (second replicate unlabeled RNA), GSM4255961 (second replicate labeled RNA), GSM4255973 (third replicate unlabeled RNA), GSM4255965 (third replicate labeled RNA). These raw data are accessible using the SRA Toolkit or another SRA downloading tool.
