# Supplementary material for: Estimating RNA dynamics using one time point for one sample in a single-pulse metabolic labeling experiment
Source: BMC Bioinformatics. 2022 Apr 22;23:147. doi: 10.1186/s12859-022-04672-4 (PMC9034570; doi:10.1186/s12859-022-04672-4)
Supplement: Supplementary file 1 — Additional file 1: Appendix A. Derivation of the model solution. [file 12859_2022_4672_MOESM1_ESM.pdf]

Appendix A Derivation of the model solution

This is a first order linear ordinary differential equation in $p(t)$ and $m(t)$ that can be expressed in matrix form as

$$\begin{pmatrix} \dot{p} \\ \dot{m} \end{pmatrix} = \begin{pmatrix} -\beta & 0 \\ \beta & -\gamma \end{pmatrix} \begin{pmatrix} p \\ m \end{pmatrix} + \begin{pmatrix} \alpha \\ 0 \end{pmatrix} \tag{A.18}$$

The solution to this equation is given by

$$\begin{pmatrix} p \\ m \end{pmatrix} = k_1 \mathbf{v} \exp(\lambda_1 t) + k_2 \mathbf{w} \exp(\lambda_2 t) + \begin{pmatrix} \frac{\alpha}{\beta} \\ \frac{\alpha}{\gamma} \end{pmatrix}, \tag{A.19}$$

where k_1 and k_2 are scalar constants determined by the boundary conditions, λ_1, λ_2 are eigenvalues of the matrix in (A.18) and $\mathbf{v}$, $\mathbf{w}$ are the corresponding eigenvectors.
The eigenvalues are given by $\lambda_1 = -\beta$ and $\lambda_2 = -\gamma$. The first eigenvector $\mathbf{v}$ is obtained by solving

$$\begin{cases} -\beta \mathbf{v}_1 = -\beta \mathbf{v}_1 \\ \beta \mathbf{v}_1 - \gamma \mathbf{v}_2 = -\beta \mathbf{v}_2 \end{cases} \Rightarrow \mathbf{v}_1 = \frac{\gamma - \beta}{\beta} \mathbf{v}_2 \Rightarrow \mathbf{v} \propto \begin{pmatrix} \gamma - \beta \\ \beta \end{pmatrix} \tag{A.20}$$

Similarly the second eigenvector is obtained by solving

$$\begin{cases} -\beta \mathbf{w}_1 = -\gamma \mathbf{w}_1 \\ \beta \mathbf{w}_1 - \gamma \mathbf{w}_2 = -\gamma \mathbf{w}_2 \end{cases} \Rightarrow \mathbf{w}_1 = 0 \Rightarrow \mathbf{w} \propto \begin{pmatrix} 0 \\ 1 \end{pmatrix}.$$

The solution to (A.18) is thus given by

$$\begin{pmatrix} p \\ m \end{pmatrix} = k_1 \begin{pmatrix} \gamma - \beta \\ \beta \end{pmatrix} \exp(-\beta t) + k_2 \begin{pmatrix} 0 \\ 1 \end{pmatrix} \exp(-\gamma t) + \begin{pmatrix} \frac{\alpha}{\beta} \\ \frac{\alpha}{\gamma} \end{pmatrix}.$$

Expressed by its component this is equivalent to

$$p(t) = k_1(\gamma - \beta)\exp(-\beta t) + \frac{\alpha}{\beta} \tag{A.21}$$

$$m(t) = k_1 \beta \exp(-\beta t) + k_2 \exp(-\gamma t) + \frac{\alpha}{\gamma} \tag{A.22}$$

We now turn to the boundary conditions to determine k_1 and k_2. The boundary conditions are different for the unlabeled and the labeled RNA.

Unlabeled RNA

Like in [19], we assume the system to be in steady-state prior to labeling. The steady-state is given by solving (A.18) with $\dot{p} = \dot{m} = 0$.

$$\begin{cases} 0 = -\beta p + \alpha \\ 0 = \beta p - \gamma m \end{cases} \Rightarrow \begin{cases} p = \frac{\alpha}{\beta} \\ 0 = \beta \frac{\alpha}{\beta} - \gamma m \end{cases} \Rightarrow \begin{cases} p = \frac{\alpha}{\beta} \\ m = \frac{\alpha}{\gamma} \end{cases} \tag{A.23}$$

During labeling time, we assume that no unlabeled RNA is synthesized such that $\alpha = 0$. Assuming that we start labeling at time $t = 0$, we thus have

$$p_u(0) = \frac{\alpha}{\beta} \Rightarrow k_1(\gamma - \beta) = \frac{\alpha}{\beta} \Rightarrow k_1 = \frac{\alpha}{\beta(\gamma - \beta)} \tag{A.24}$$

Moreover we have

$$m_u(0) = \frac{\alpha}{\gamma} \Rightarrow \frac{\alpha}{\gamma - \beta} + k_2 = \frac{\alpha}{\gamma} \Rightarrow k_2 = \frac{\alpha}{\gamma} - \frac{\alpha}{\gamma - \beta} = \frac{-\beta\alpha}{\gamma(\gamma - \beta)}$$

This leads us to the solution for the unlabeled RNA

$$p_u(t) = \frac{\alpha}{\beta} \exp(-\beta t) \tag{A.25}$$

$$m_u(t) = \frac{\alpha}{\gamma - \beta} \exp(-\beta t) - \frac{\beta\alpha}{\gamma(\gamma - \beta)} \exp(-\gamma t)\,, \tag{A.26}$$

where the u label indicates that this corresponds to the unlabeled RNA pool.

Labeled RNA

The solution for the labeled RNA could be obtained the same way as for the unlabeled RNA, but setting $\alpha \neq 0$ and $p_l(0) = m_l(0) = 0$. However, it is simpler to notice that the total RNA (labeled and non-labeled) stay at steady-state during the labeling such that we have the following solution for labeled RNA.

$$p_l(t) = \frac{\alpha}{\beta} - p_u(t) = \frac{\alpha}{\beta}\big(1 - \exp(-\beta t)\big)$$

$$m_l(t) = \frac{\alpha}{\gamma} - m_u(t) = \frac{\alpha}{\gamma}\big(1 + \frac{\beta}{(\gamma - \beta)} \exp(-\gamma t)\big) - \frac{\alpha}{\gamma - \beta} \exp(-\beta t)$$

where the l label indicates that this corresponds to the labeled RNA pool.
