# Supplementary material for: Estimating RNA dynamics using one time point for one sample in a single-pulse metabolic labeling experiment
Source: BMC Bioinformatics. 2022 Apr 22;23:147. doi: 10.1186/s12859-022-04672-4 (PMC9034570; doi:10.1186/s12859-022-04672-4)
Supplement: Supplementary file 2 — Additional file 2: Appendix B. Equation simplification. [file 12859_2022_4672_MOESM2_ESM.pdf]

Appendix B Equation simplification

In this appendix we show how the the system given by equations (10, 11) can be simplified to Eq. (13) to infer the ratio k between the processing and degradation rate. Starting from

$$a = \frac{(1-k)E_{k\gamma}}{E_{k\gamma} - k^2 E_\gamma} \tag{B.27}$$

$$b = \frac{(1-k)\big(1-E_{k\gamma}\big)}{\big(1-E_{k\gamma}\big) - k^2\big(1-E_\gamma\big)}\,, \tag{B.28}$$

we have

$$a\Big(E_{k\gamma} - k^2 E_\gamma\Big) = (1-k)E_{k\gamma} \tag{B.29}$$

$$b\Big(\big(1-E_{k\gamma}\big) - k^2(1-E_\gamma)\Big) = (1-k)\big(1-E_{k\gamma}\big)\,. \tag{B.30}$$

Summing (B.29) and (B.30) yields

$$E_{k\gamma}(a-b) + k^2 E_\gamma(b-a) + b(1-k^2) = 1-k \tag{B.31}$$

$$\begin{aligned} \Leftrightarrow \quad E_{k\gamma} - k^2 E_\gamma &= \frac{(1-k) - b(1-k^2)}{a-b} \\ &= \frac{(1-k)\big(1-b(1+k)\big)}{a-b}\,. \end{aligned} \tag{B.32}$$

Dividing (B.29) by (B.30) and inserting (B.32) results in

$$\begin{aligned} \frac{E_{k\gamma}}{1-E_{k\gamma}} &= \frac{a}{b}\frac{E_{k\gamma} - k^2 E_\gamma}{\big(1-E_{k\gamma}\big) - k^2(1-E_\gamma)} \\ &= \frac{a}{b}\frac{E_{k\gamma} - k^2 E_\gamma}{(1-k^2) - \big(E_{k\gamma} - k^2 E_\gamma\big)} \\ &= \frac{a}{b}\frac{(1-k)(1-b(1+k)}{(1-k^2)(a-b) - (1-k)\big(1-b(1+k)\big)} \\ &= \frac{a}{b}\frac{1-b(1+k)}{(1+k)(a-b) - 1 + b(1+k)} \\ &= \frac{a}{b}\frac{1-b(1+k)}{(1+k)a-1} = -\frac{a-ab(1+k)}{b-ab(1+k)} \end{aligned} \tag{B.33}$$

It follows that

$$E_{k\gamma}\big(b - ab(1+k)\big) = \big(E_{k\gamma} - 1\big)\big(a - ab(1+k)\big) \tag{B.34}$$

$$\Leftrightarrow \quad (b-a)E_{k\gamma} = ab(1+k) - a\,, \tag{B.35}$$

an thus

$$\exp(-k\gamma T) = E_{k\gamma} = \frac{kab + ab - a}{b-a} = \frac{a(bk + b - 1)}{b-a} \tag{B.36}$$

$$\Leftrightarrow \quad k\gamma T = \log\big(\frac{b-a}{a(bk+b-1)}\big) \tag{B.37}$$

Moreover, from (10), we have that

$$a = \frac{1-k}{1-k^2 \exp\big((k-1)\gamma T\big)} \Leftrightarrow \exp\big((k-1)\gamma T\big) = \frac{(1-\frac{1-k}{a})}{k^2}$$
$$\Leftrightarrow \quad (k-1)\gamma T = \log\big(\frac{k+a-1}{k^2 a}\big) \tag{B.38}$$

Multiplying (B.37) by $\frac{k-1}{k}$ and subtracting (B.38) results in

$$0 = \frac{k}{k-1}\log\big(\frac{k+a-1}{k^2 a}\big) - \log\Big(\frac{(b-a)}{a(bk+b-1)}\Big). \tag{B.39}$$

This equation also provides upper and lower bounds for k as both $\frac{k+a-1}{a}$ and $bk+b-1$ must be strictly positive for their logarithm to be defined and

$$0 < \exp(-\beta T) = E_{k\gamma} = \frac{kab+ab-a}{b-a} < 1 \quad \forall \beta T > 0 \tag{B.40}$$

for (B.36) to hold. Developing these three conditions results in the following domain of definition $\mathcal{D}$ for k:

$$\max(\frac{1}{b}-1, 1-a) < k < \frac{1}{a}-1\,, \tag{B.41}$$

where $0 < a < b < 1$.
