# Supplementary material for: Estimating RNA dynamics using one time point for one sample in a single-pulse metabolic labeling experiment
Source: BMC Bioinformatics. 2022 Apr 22;23:147. doi: 10.1186/s12859-022-04672-4 (PMC9034570; doi:10.1186/s12859-022-04672-4)
Supplement: Supplementary file 3 — Additional file 3: Appendix C. Proof of uniqueness of solution. [file 12859_2022_4672_MOESM3_ESM.pdf]

Appendix C Proof of uniqueness of solution

In this appendix, we prove that (13) has a single solution for $b > (2-a)^{-1}$. We first note that $b > (2-a)^{-1} \Leftrightarrow \frac{1-b}{b} < 1-a$, so the lower bound for k is $k_- = 1-a$. We then define the right-hand side of (13) as

$$\begin{aligned} g(x) &= \frac{x}{x-1} \log\left(\frac{1}{x^2}(1-\frac{1-x}{a})\right) - \log\left(\frac{(b-a)}{a\big(b(x+1)-1\big)}\right) && \text{(C.42)} \\ &= \frac{x}{x-1}\Big(-2\log(x) + \log(a+x-1) - \log(a)\Big) \\ &\quad - \log(b-a) + \log(a) + \log\big(b(x+1)-1\big) && \text{(C.43)} \end{aligned}$$

We then observe that on that lower bound $\lim_{x \overset{>}{\to} 1-a} g(x) = +\infty$ because $a+x-1$ tends to zero and $x-1$ is negative.
On the other hand, for the upper bound $k_+ = \frac{1-a}{a}$, we have

$$\begin{aligned} a+x-1 &= \frac{a^2+1-a-a}{a} = \frac{(1-a)^2}{a} \\ b(x+1)-1 &= \frac{b}{a} - 1 = \frac{b-a}{a} \end{aligned}$$

we can deduce that the upper bound of x is a zero of g:

$$\begin{aligned} & g(\frac{1-a}{a}) = \\ & \frac{1-a}{1-2a}\Big(-2\log(1-a) + 2\log(a) + 2\log(1-a) - 2\log(a)\Big) \\ & -\log(b-a) + \log(a) + \log(b-a) - \log(a) = 0. && \text{(C.44)} \end{aligned}$$

Moreover, the derivative of g is given by:

$$\begin{aligned} g'(x) = &-\frac{1}{(x-1)^2}\big(-2\log(x) + \log(a+x-1) - \log(a)\big) \\ &+ \frac{x}{x-1}\big(-\frac{2}{x} + \frac{1}{a+x-1}\big) + \frac{b}{b(x+1)-1}. && \text{(C.45)} \end{aligned}$$

Then

$$\begin{aligned} g'(\frac{1-a}{a}) =& \frac{-a^2}{(1-2a)^2}\big(-2\log(1-a) + 2\log(a) + 2\log(1-a) \\ & -2\log(a)\big) + \frac{1-a}{1-2a}\big(\frac{-2a}{1-a} + \frac{a}{(1-a)^2}\big) + \frac{ba}{b-a} \\ =& \frac{1}{1-2a}\frac{2a-1}{1-a} + \frac{ba}{b-a} \\ =& -\frac{a}{1-a} + \frac{ba}{b-a} \\ =& \frac{a^2(1-b)}{(b-a)(1-a)} > 0 \quad \text{if } 0 < a < b < 1. && \text{(C.46)} \end{aligned}$$

Since $g(k_+)$ reaches zero from below, while $g(k_-) > 0$, we can infer that $g(x)$ has a zero between k_- and k_+ as illustrated on Fig C.7.

To show that this zero is unique, we look at the sign of $g'(x)$.

We can rewrite $g'(x)$ as

$$g'(x) = \frac{1}{(x-1)^2}\big(A(x) - B(x)\big) \tag{C.47}$$

where

$$A(x) = \frac{(1-x)\big(x(ab+b-1) - (3b-2)(1-a)\big)}{(x+a-1)(bx+b-1)}\Big)$$
$$B(x) = \log\big(\frac{a+x-1}{ax^2}\big)$$

Let x_0 be a zero of g', i.e., the position of a local extrema of g. We have

$$\begin{aligned} g(x_0) &= \frac{x_0}{x_0-1}\log\big(\frac{x_0+a-1}{ax_0^2}\big) - \log\Big(\frac{(b-a)}{a\big(b(x_0+1)-1\big)}\Big) \\ &= \frac{-x_0\big(x_0(ab+b-1) - (3b-2)(1-a)\big)}{(x_0+a-1)(bx_0+b-1)} \\ &\quad - \log\Big(\frac{(b-a)}{a\big(b(x_0+1)-1\big)}\Big) \end{aligned} \tag{C.48}$$

The second equality holds because $g'(x_0) = 0$ by definition of x_0. By multiplying (C.48) by $(bx_0 + b - 1)$, which is positive, we can then define a new function $h(x)$ whose sign is the same as the sign of $g(x)$ for $x = x_0$ (see Fig C.7 for an illustration).

$$h(x) = C(x) - D(x)\,, \tag{C.49}$$

where

$$\begin{aligned} C(x) &= \frac{-x\big(x(ab+b-1) - (3b-2)(1-a)\big)}{(x+a-1)} \\ D(x) &= (bx+b-1)\log\Big(\frac{(b-a)}{a\big(bx+b-1\big)}\Big) \end{aligned} \tag{C.50}$$

We can now compute the second derivatives of $C(x)$ and $D(x)$.

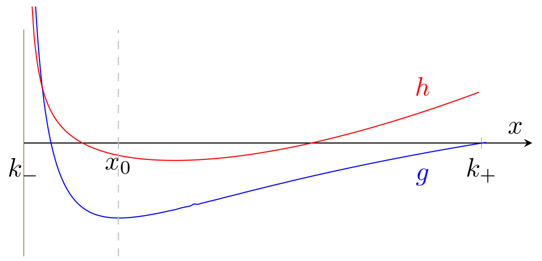

Figure C.7: Sketch of the proof that $g(x)$ has a single zero in $\mathcal{D} = (k_-, k_+)$. We first show that $\lim_{x\to k_-} g(x) = \infty$, that $\lim_{x\to k_+} g(x) = 0$ and $\lim_{x\to k_+} g'(x) > 0$, so that g must cross the x-axis on $\mathcal{D}$. To show that it only does it once, we consider a function $h(x)$ that has the same sign as $g(x)$ when $g'(x) = 0$. We show that h is convex on $\mathcal{D}$ and thus g cannot have a negative extrema, followed by a positive extrema, followed by a negative extrema. Hence it cannot have more than one zero on $\mathcal{D}$.

$$
\begin{aligned}
C'(x) =& -\frac{\big(2x(ab+b-1)-(3b-2)(1-a)\big)(x+a-1)}{(x+a-1)^2} \\
&+\frac{x^2(ab+b-1)-x(3b-2)(1-a)}{(x+a-1)^2} \\
=& -\frac{x^2(ab+b-1)-2x(1-a)(ab+b-1)+(3b-2)(1-a)^2}{(x+a-1)^2} \\
C''(x) =& -\frac{\big(2x(ab+b-1)-2(1-a)(ab+b-1)\big)(x+a-1)}{(x+a-1)^3} \\
&+\frac{2\big(x^2(ab+b-1)-2x(1-a)(ab+b-1)+(3b-2)(1-a)^2\big)}{(x+a-1)^3} \\
=& -\frac{2(1-a)^2(ab-2b+1)}{(x+a-1)^3} > 0 \quad \forall b > \frac{1}{2-a} \\
D'(x) =& b\log\big(\frac{b-a}{a}\big) - b\big(\log(bx+b-1)+1\big) \\
D''(x) =& -\frac{b^2}{bx+b-1} < 0 \quad \forall x > k_-
\end{aligned}
$$

Hence

$$
h''(x) = C''(x) - D''(x) < 0 \quad \forall x \in \mathcal{D}, \forall b > \frac{1}{2-a} \tag{C.51}
$$

This means that h is convex, so there cannot be three points $x_1 < x_2 < x_3$ such that $0 > h(x_1) < h(x_2) > 0 > h(x_3)$. Hence the same can be said of three zeros of g', so $g(x)$ cannot have more that one zero. $\square$
