# Supplementary material for: Estimating RNA dynamics using one time point for one sample in a single-pulse metabolic labeling experiment
Source: BMC Bioinformatics. 2022 Apr 22;23:147. doi: 10.1186/s12859-022-04672-4 (PMC9034570; doi:10.1186/s12859-022-04672-4)
Supplement: Supplementary file 4 — Additional file 4: Appendix D. Supplementary figures. [file 12859_2022_4672_MOESM4_ESM.pdf]

Appendix D Supplementary figures

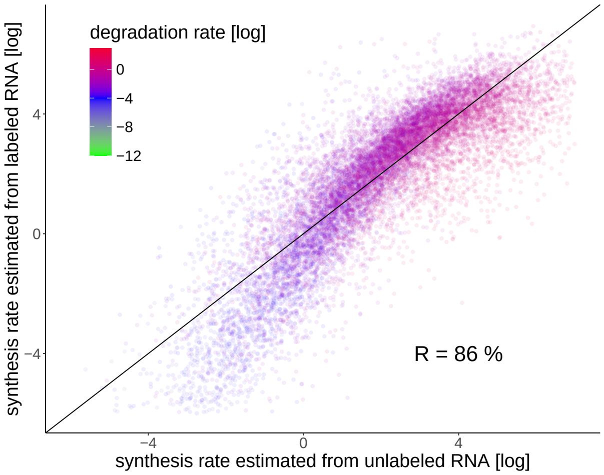

Figure D.8: Correlation of synthesis rates derived from the unlabeled and the labeled RNA pools is good at 86%. "Slow" transcripts, with low synthesis and degradation rates (in blue) tend to show a more systematic difference between the two estimates. This can be explained by the fact that those transcripts are likely to be more affected by residual unlabeled RNA captured in the labeled RNA pool through unspecific binding. Only transcripts with an exact solution to (13) were considered.

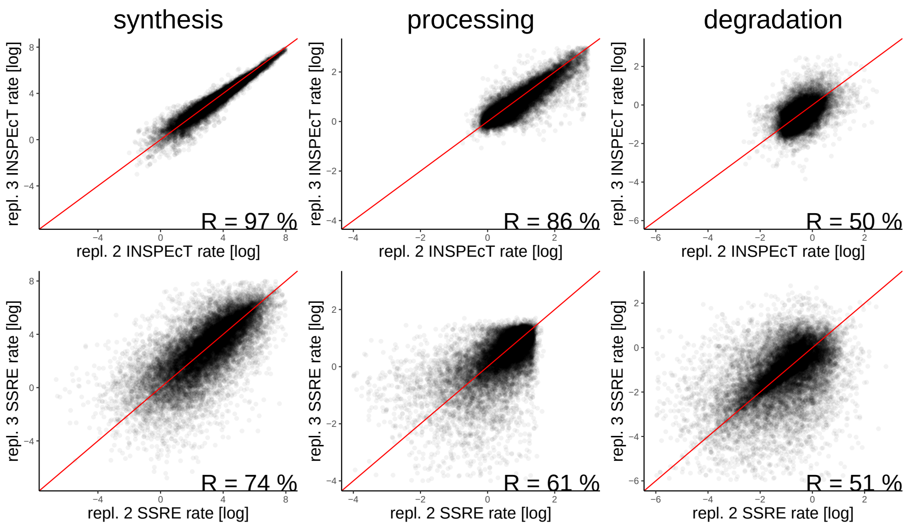

Figure D.9: Correlation of rates across two replicates. Top row: Rates obtained with INSPEcT. Bottom row: rates obtained with our method. The INSPEcT method provides rates that are more consistent across replicates for synthesis and processing rates, but not for degradation rates. Spearman correlation is indicated and the red line shows the diagonal.
